# To Stop the Secretion of Milk

**Published:** 1890-03

**Authors:** 


TO STOP THE SECRETION OF MILK.—Dissolve one-half ounce of camphor in twelve ounces of turpentine, and apply to the breasts when desiring to stop the secretion of milk.—*Exchange.*

---

A young physician was showing a friend a recent purchase he had made in the way of a skeleton. "Very interesting," commented his friend. "One of your patients, doctor?"

